# Beyond the Urogenital Tract, the Role of *Ureaplasma parvum* in Invasive Infection in Adults: A Case Series and Literature Review

**DOI:** 10.3390/diagnostics15172242

**Published:** 2025-09-04

**Authors:** Linhui Hu, Xiangyan Li, Dan Liu, Jie Yao, Xueying Li, Yan Wang

**Affiliations:** Department of Infectious Disease, Peking University First Hospital, Beijing 100034, China; hlh1569780519@163.com (L.H.); lixyan03598@pkufh.com (X.L.); 13683310202@163.com (D.L.); yjjel13601017514@163.com (J.Y.); l13301119117@163.com (X.L.)

**Keywords:** *Ureaplasma parvum*, urogenital tract, infection, next-generation sequencing

## Abstract

**Background/Objectives**: *Ureaplasma parvum* (*Up*) is an opportunistic pathogen associated with urogenital tract infections, pregnancy complications, and reproductive system diseases. Advances in molecular diagnostics have expanded its pathogenic spectrum to include invasive conditions such as arthritis, meningitis, and pneumonia. However, the pathogenic significance of *Up* remains controversial. **Methods**: This study retrospectively analyzed nine adult cases of *Up* detected by metagenomic next-generation sequencing (mNGS) between 2023 and 2024. **Results**: Patients, aged 21 to 70 years, predominantly had underlying immunosuppressive conditions (66.7%). Infections involved the urinary system (44.4%), respiratory system (33.3%), and peritoneal cavity (22.2%). Symptomatic relief was achieved in five cases following treatment with tetracyclines, quinolones or tigecycline. **Conclusions**: These findings highlight *Up* as a potential causative agent of invasive infections, particularly in immunocompromised patients. *Up* has potential pathogenic significance, whether it is detected as a single pathogen or as a coexisting pathogen.

## 1. Introduction

Globally, *Ureaplasma* spp. colonizes 40–80% of asymptomatic American women, 8.9% of young Norwegians, and 20–47% of symptomatic patients in the United States, China, and Greece [1]. *Ureaplasma parvum* (*Up*), a member of the Family Mycoplasmataceae and the genus *Ureaplasma* spp., is characterized by its unique biological features: the absence of a cell wall, the dependence on host urease for metabolic activity, and the capacity for vertical transmission. *Up* typically functions as an opportunistic pathogen, frequently colonizing the mucosal surfaces of the urogenital tract [2]. Recent studies have established the significant pathogenicity of *Up* in pediatric infections and urogenital diseases in adults. In neonates, *Up* can cause pneumonia, meningitis, and bronchopulmonary dysplasia through vertical transmission from mother to child [3,4,5]. In women of childbearing age, *Up* colonization is closely associated with pregnancy complications such as spontaneous abortion, premature rupture of membranes, and preterm labor [6,7]. While in men, it has been related to urethritis and infertility [8,9].

With the widespread application of molecular diagnostic techniques in these years, such as metagenomic next-generation sequencing (mNGS), the detection of *Up* in adult infections has expanded beyond the urogenital system. Reports suggested that *Up* may be involved in invasive diseases such as septic arthritis, meningitis, pneumonia, and peritoneal infections [10,11,12,13]. However, most *Up* colonized individuals do not develop clinical disease. This has generated considerable debate regarding its pathogenic role in adult non-urogenital infections, specifically whether *Up* functions as a true pathogen or merely represents incidental detection of a commensal organism. Answering this question is crucial for precise clinical diagnosis and treatment. Based on this background, this study retrospectively analyzed adult infection cases detected *Up* by mNGS—from January 2023 to December 2024 at Peking University First Hospital, a national tertiary referral and academic medical center in Beijing, China. We systematically summarized the clinical characteristics, infection spectrum, and treatment outcomes of these cases. Relevant studies were also reviewed to explore the pathogenic significance of *Up* in adult non-genitourinary infections, as well as to reassess its pathogenic potential through molecular diagnostic techniques. By integrating multidimensional evidence, we aim to provide new insights into the clinical significance of *Up*.

## 2. Methods

This study employed a retrospective review of medical records to investigate the clinical characteristics, microbiological findings, and treatment outcomes of patients with *Up* detected by mNGS. This study was conducted in accordance with the Declaration of Helsinki and was approved by the Institutional Review Board (IRB) of Peking University First Hospital (Approval No. 2025-078-001), which waived the requirement for individual informed consent.

### 2.1. Patients Selection and Data Collection

This study included nine adult patients who tested positive for *Up* by mNGS between January 2023 and December 2024. The inclusion criteria were as follows: (1) positive mNGS results for *Up*; (2) age ≥ 18 years.

Demographic and clinical data were extracted from the electronic medical records, including age, gender, comorbidities, clinical symptoms, and infection site. Underlying conditions were categorized as immunosuppressive or non-immunosuppressive based on the presence of malignancies, diabetes mellitus, kidney injury, peritoneal dialysis, or obesity.

### 2.2. Specimen Collection and Next-Generation Sequencing

A total of 9 clinical specimens were analyzed using mNGS, including urine, blood, sputum, and bronchoalveolar lavage fluid (BALF) samples. Specimens were collected according to standard clinical protocols and processed for nucleic acid extraction. Library preparation was performed by using an NGS Automatic Library Preparation System (MatriDx Biotech Corp., Hangzhou, China). The quality of DNA was assessed using a BioAnalyzer 2100 (Agilent Technologies; Santa Clara, CA, USA) combined with quantitative PCR to measure the adapters before sequencing. Each sample generated a total of 0.1–1 million reads. A sequencing depth of 0.1–1 million reads per sample was adopted owing to host nucleic acid depletion, which reduces human-derived sequences by over two orders of magnitude. This enrichment of microbial reads improves pathogen detection, meets clinical diagnostic standards, and lowers sequencing demand, providing cost-effective benefits for routine application. Raw sequenced reads underwent quality control processing to eliminate short (length < 35 bp), low-quality, and low complexity reads, along with the sequencing adapters. Sequences from the host were excluded by aligning them to the human-specific database in NCBI (GRCh38.p13), utilizing Bowtie2 (version 2.3.5.1, https://sourceforge.net/projects/bowtie-bio/files/bowtie2/2.3.5.1/bowtie2-2.3.5.1-linux-x86_64.zip, accessed on 30 June 2025). Kraken2 (source code, https://github.com/DerrickWood/kraken2, accessed on 30 June 2025) was used for rapid classification, followed by secondary alignment with Bowtie2 to enhance classification accuracy. When inconsistencies arose between the results of Kraken2 and Bowtie2, the classification of reads was determined using BLAST (version 2.9.0, ftp://ftp.ncbi.nlm.nih.gov/blast/executables/blast+/2.9.0/ncbi-blast-2.9.0+-src.tar.gz, accessed on 30 June 2025). The numbers of reads per one million sequencing reads were calculated. Microbial reads from a library were reported if: (1) the sequencing data met specific quality control thresholds (library concentration > 1 pM, Q20 > 85%, Q30 > 80%); (2) the negative control in the same sequencing run either did not contain the species or the reads per one million sequencing reads ratio (sample to negative control) was ≥5, a cutoff established for differentiating true positives from background contaminates [14,15]. The positive control comprised a synthetic mixture of an RNA virus (*influenza A*), a DNA virus (*Epstein–Barr virus*), a bacterium (*Klebsiella pneumoniae*), and a fungus (*Candida albicans*), validating assay performance across pathogen classes. Reagent-derived contaminants were assessed with negative controls, and only taxa detected in samples but absent from controls were considered clinically relevant.

mNGS analyses were performed, with the aim of identifying microbial pathogens (bacteria, viruses, fungi, and other microorganisms) and quantifying their relative abundances and sequence number. The relative abundance and sequence number of *Up* was recorded for each positive sample, and co-existing microbial species were also documented.

### 2.3. Treatment and Outcome Assessment

Treatment strategies were determined based on the clinical judgment of the treating physicians, with some patients receiving targeted antibiotic therapy based on the mNGS results. The antibiotics used included tetracyclines and quinolones. Treatment outcomes were assessed based on the resolution of clinical symptoms and persistence of infections. Complete symptom resolution was defined as the absence of fever, pain, or other infection-related symptoms within 2 weeks of follow-up. Persistent infections were defined as unresolved symptoms or sustained positive microbiological findings following initial treatment.

### 2.4. Statistical Analysis

All data are presented individually in tabular form. The exact value for each patient is listed; values that required anonymization are provided as ranges. Figure was generated with the built-in charting tools in Microsoft Office.

## 3. Results

**Figure 1:**
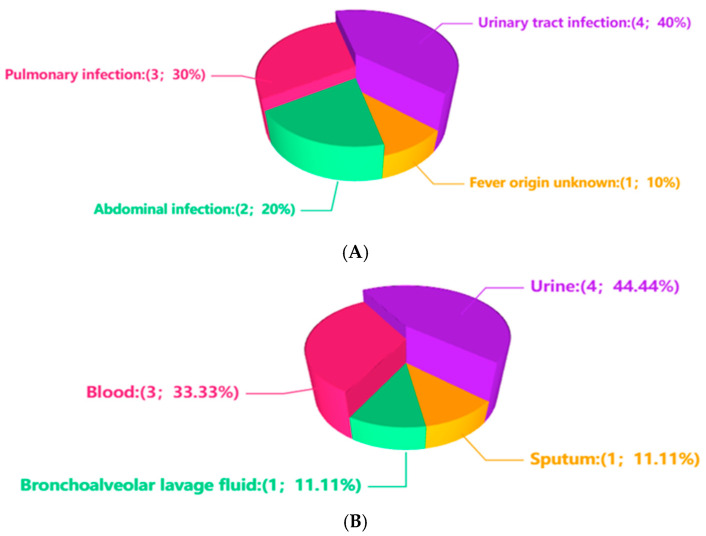
Infection sites and specimen types. (**A**) shows the infection site among the 9 cases. (**B**) shows the specimen type among the 9 samples.

As shown in Figure 1A, among these cases, 8 patients were confirmed to have infections by clinicians, including 4 cases of urinary tract infection, 2 cases of pulmonary infection, 1 case of peritoneal dialysis-associated peritonitis, and 1 case of multisite infection involving both pulmonary and abdominal system. The remaining patient was diagnosed with fever of unknown origin. The patients ranged in age from 21 to 70 years, with 5 females (55.6%) and 4 males (44.4%). 6 patients (66.7%) had underlying immunosuppressive conditions, including malignancies, acute or chronic renal failure, diabetes mellitus, one undergoing peritoneal dialysis. A total of 9 clinical specimens were analyzed by mNGS, comprising 4 urine samples, 3 blood samples, 1 sputum sample, and 1 BALF sample (Figure 1B). The relative abundance of *Up* ranged from 0.03% to 31.56%. Co-existing microbial species and their relative abundances are detailed in Table 1.

Case 1 was a 70-year-old elderly woman hospitalized for “non-Hodgkin’s lymphoma”. During her hospital stay, she developed fever with a rash. Laboratory and imaging tests revealed no significant abnormalities. *Up* was detected in her blood sample by mNGS, with a relative abundance of 0.68%. Patient in case 2 was an overweight young man with a diagnosis of fever of unknown origin. His mNGS detected only *Up*, with a relative abundance of 0.81%. He did not revisit our hospital for fever within two weeks. Case 3 presented with dyspnea and chest pain. Laboratory results indicated a mild increase in white blood cells (WBC). The patient was also found to have *mycobacterium tuberculosis complex*, with a relative abundance of 2.48%, while the relative abundance of *Up* was 0.06%. Patient in case 5 was a young woman undergoing long-term peritoneal dialysis, with underlying diabetes and hypertension. She presented with abdominal pain, and laboratory tests showed a percentage of neutrophils (NE%) of 89.7% and C-reactive protein of 197.21 mg/L. Subsequently, mNGS was performed on her peripheral blood, which indicated an infection with *Up* and *Cytomegalovirus (CMV)*, with a relative abundance of *Up* and *CMV* both at 7.14%. Case 8 involved a middle-aged man with a malignant retroperitoneal pheochromocytoma. His initial lab results showed significantly elevated WBC and NE%. mNGS detected *Pseudomonas aeruginosa* (*PA*, 77.04%) and *Up* (0.03%).

Regarding treatment and prognosis, 5 patients (Cases 4, 5, 6, 7, and 9) received antibiotics targeting *Up* (3 treated with minocycline, 1 with tigecycline and 1 with moxifloxacin), achieving complete symptom resolution. 1 patient (Case 2) tested positive for *Up* but did not receive treatment. Another patient (Case 3), who tested positive for *mycobacterium tuberculosis* and *Up*, received anti-*tuberculosis* treatment outside the hospital. The remaining 2 patients did not receive targeted anti-infective treatment. Among them, Case 1 had persistent fever for more than 2 weeks. Case 8 improved within 2 weeks after receiving treatment targeting *PA*.

## 4. Discussion

In this case series, *Up* was detected in all nine patients by mNGS, emphasizing the diverse clinical manifestations and infection sites that expand current understanding of its pathogenic characteristics. To differentiate true infection from colonization when *Up* was detected, infectious-disease specialists systematically weighed mNGS data against each patient’s clinical context and therapeutic response. There are four patients in this series presented with urinary tract infections. Notably, *Up* was also isolated from blood, sputum, and BALF samples in patients diagnosed with abdominal infections (22.2%, 2/9) and pneumonia (33.3%, 3/9). Although distinguishing colonization from true infection remains critical, these findings underscore *Up*’s potential to cause multisystem infections, a phenomenon supported by emerging evidence in the literature.

*Up* is well-established as a common colonizer of the urogenital tract, with its primary clinical association being urogenital infections. Noma et al. demonstrated that *Up*—when co-detected with high-risk *human papillomavirus*—significantly elevates the risk of low-grade squamous intraepithelial lesions in the cervix [16]. Furthermore, *Up* is an established pathogen of urethritis, which is associated with urethral inflammation severity and its capacity to disrupt sperm quality, contributing to impaired fertility [8,9,17]. Bacterial loads of *Up* contribute to the development of inflammatory responses in the male urethra. In our study, case 4, 5, 6, 7, and 9 were those who improved after targeted treatment. Among them, case 4, 6, 7, and 9 had urinary tract infections, and their symptoms and/or inflammatory indicators were alleviated within two weeks after targeted treatment for *Up*. Therefore, our study further affirms the pathogenicity of *Up* in the urogenital tract.

Beyond its urogenital tropism, a growing body of evidence has expanded the recognized pathogenic scope of *Up*. Previous studies have reported *Up* as a causative agent of purulent arthritis [10], central nervous system infections [11], and rare presentations such as hyperammonemia [18]. In our study, we identified four patients with non-urogenital tract infections. Among them, three patients were diagnosed with pulmonary infections. In case 1, pneumonia was suspected based on her clinical symptoms. However, no targeted anti-infective therapy was administered, and her fever persisted for 46 days before resolving. This raises the possibility that failure to target *Up* contributed to the prolonged febrile episode. In case 5, the patient was diagnosed with an abdominal infection and treated with tigecycline. Within two weeks, her C-reactive protein decreased from 197.21 to 47.07 mg/L, NE% dropped from 89.7% to 53.8%, and her abdominal pain was alleviated, suggesting that *Up* played a significant role in her peritonitis. Collectively, these observations support the hypothesis that *Up* may represent a pathogen of clinical significance.

However, at the same time, cases 2, 3, and 8 also remind us that not all patients with *Up* detected by mNGS have pathogenic significance. We need to consider its relative abundance and clinical manifestations. In case 3, the patient was diagnosed with a pulmonary infection, and mNGS detected both *mycobacterium tuberculosis complex* and *Up*. After two weeks of anti-*tuberculosis* treatment outside the hospital, the patient did not return for follow-up due to persistent fever. In case 8, mNGS detected *PA* and *Up;* clinically, his pneumonia and peritoneal infection were attributed to *PA*. He was treated with ceftazidime-avibactam, teicoplanin, and inhaled amikacin, resulting in a marked decrease in WBC count and NE% within two weeks. Based on these observations, we conclude that *Up* is unlikely to be pathogenically relevant in these two cases.

Although *Up* infections have been sporadically reported in immunocompetent individuals [19,20], immunocompromised populations (e.g., patients with malignancies or post-transplantation) exhibit heightened susceptibility [11,18], as evidenced by the 66.7% (6/9) prevalence of immunosuppression in this case series. More importantly, among the five patients in whom *Up* was detected and who received targeted treatment, three were immunocompromised. Notably, these individuals showed significant improvement in both symptoms and inflammatory markers following treatment. These findings underscore the potential of *Up* as a pathogen responsible for invasive infections, particularly in immunosuppressed populations. Clearly defining targeted therapeutic strategies for *Up* infection is therefore of critical importance in improving clinical outcomes for immunocompromised patients.

Previous reports have confirmed the pathogenicity of *Up* as a single pathogen [21], a finding corroborated by case 6 in our series, where symptomatic resolution followed moxifloxacin therapy targeting *Up*, the sole pathogen detected. Notably, *Up* was identified alongside *Mycobacterium tuberculosis complex*, *CMV*, and *Pseudomonas aeruginosa* in BALF, sputum, and peripheral blood samples in our study. This aligns with Rehman et al.’s report of *Up* coexisting with *multidrug-resistant PA*, *Haemophilus influenzae*, *human coronaviruses*, *Mycoplasma pneumoniae*, and *CMV* in respiratory samples [22]. Although the clinical dominance of *Up* in such polymicrobial infections remains unclear, the therapeutic response to *Up*-targeted antimicrobials in our cases 4, 5, 7, and 9 provides compelling evidence of its pathogenic contribution. Specifically, in cases 4, 5, and 9, the relative abundance of *Up* was found to be lower than or equal to that of the coexisting pathogens. This suggests that *Up* may still play a significant role in the infection, even in the presence of other coinfecting organisms.

The precise mechanisms driving *Up* infections remain incompletely elucidated but are hypothesized to involve dysregulated activation of innate immunity. Notably, *Up* membrane lipoproteins stimulate the NF-κB pathway through TLR2/6/9 receptors, triggering a pro-inflammatory cytokine storm characterized by elevated IL-6 and TNF-α [23]. This distinct immunostimulatory profile may explain *Up*’s tropism for TLR-rich tissues and its ability to invade the central nervous system post–blood–brain barrier compromise, though mechanisms in other systems warrant further exploration.

Diagnosing *Up* infections presents dual challenges: conventional culture frequently fails due to the organism’s fastidious growth requirements, while asymptomatic colonization complicates clinical differentiation. Advances in molecular diagnostics, particularly mNGS, have revolutionized the field by enabling rapid and highly sensitive detection of pathogens. In this series, mNGS identified *Up* in custom culture-negative specimens, underscoring the indispensable role of molecular methods. For sterile sites, detection of *Up* via mNGS strongly suggests pathogenicity. Conversely, in colonization-prone regions (e.g., respiratory tract), interpretation requires integration of bacterial load, host immunity, and clinical context.

Therapeutic management of *Up* infections is complicated by its lack of a cell wall and inability to synthesize folate, rendering β-lactams, glycopeptides, sulfonamides, and diaminopyrimidines ineffective. The treatment of choice comprises tetracyclines, macrolides, and fluoroquinolones; however, rising resistance rates to these classes are increasingly documented [24]. In this series, tetracyclines and fluoroquinolones were prioritized based on *Up*’s biological susceptibility profile, achieving satisfactory short-term clinical outcomes. Notably, in central nervous system infections, agents with blood–brain barrier penetration (e.g., doxycycline combined with moxifloxacin) are preferable, as evidenced by their use in resolving *Up*-associated brain abscess [25].

While this study is limited by its retrospective design, small cohort size, and absence of comprehensive antimicrobial susceptibility profiles, it offers novel insights into *Up* as an emerging multisystem pathogen and demonstrates the superior diagnostic sensitivity of mNGS. These findings provide actionable data to inform clinical decision-making, particularly in atypical infection contexts. To advance understanding, future multicenter studies should address three critical gaps: (1) establishing site-specific diagnostic thresholds to differentiate *Up* colonization from true infection; (2) clarifying mechanistic links between immunosuppression and invasive *Up* disease; and (3) formulating precision treatment guidelines informed by molecular susceptibility testing. In summary, the optimal management of *Up* hinges on the integration of patients’ clinical case records with personalized antimicrobial regimens, especially for immunocompromised hosts.

## 5. Conclusions

Drawing on nine well-documented cases of adult invasive infection and an exhaustive literature synthesis, we demonstrate that *Up* is no longer confined to its historical niche as a mere urogenital commensal. The organism can act alone or with co-pathogens to produce clinically significant disease—particularly in immunocompromised hosts. Yet fundamental questions remain; integrated studies are urgently required to reposition *Up* from an ambiguous colonizer to an unequivocally actionable invasive pathogen.

## Figures and Tables

**Table 1:** Clinical characteristics, detected pathogens and prognosis in 9 cases.

Case	Genda	Age	Infection Sites	Specimen Types	Relative Abundance of *Up*%	Sequence Number of *Up*	Target Therapy	Prognosis
1	female	70–80	Pulmonary infection	blood	<1	≤1	No	Unresolved infection
2	male	20–30	Fever origin unknown	blood	<1	≤1	No	Unclear
3	male	40–50	Pulmonary infection	BALF	<1	1–100	No	Unclear
4	female	40–50	Urinary tract infection	urine	1–10	100–1000	Minocycline	Relieved
5	female	30–40	Abdominal infection	blood	1–10	≤1	Tigecycline	Relieved
6	male	20–30	Urinary tract infection	urine	>10	2000–3000	Moxifloxacin	Relieved
7	female	40–50	Urinary tract infection	urine	1–10	1000–2000	Minocycline	Relieved
8	male	50–60	Pulmonary infection, Abdominal infection	Sputum	<1	1–100	No	Relieved
9	female	20–30	Urinary tract infection	urine	<1	100–1000	Minocycline	Relieved
Case	Immunosuppressed individuals		Other microbial species		Relative Abundance of other microbial species(%)		Sequence number of other microbial species	
1	Yes		None		None		None	
2	No		None		None		None	
3	Yes		*Mycobacterium tuberculosis complex*		1–10		2000–3000	
4	No		*Lactobacillus crispatus*		>10		70,000–80,000	
5	Yes		*Cytomegalovirus*		1–10		≤1	
6	Yes		None		None		None	
7	Yes		*Acinetobacter junii*		<1		1–100	
8	Yes		*PA*		>10		90,000–100,000	
9	No		*Enterococcusfaecalis*		1–10		1000–2000	

Note: “20–30” indicates an age range of 20–30 years; “30–40” indicates an age range of 30–40 years; “40–50” indicates an age range of 40–50 years; “50–60” indicates an age range of 50–60 years; “70–80” indicates an age range of 70–80 years. “<1 (Relative abundance)” indicates a relative abundance < 1; “1–10 (Relative abundance)” indicates a relative abundance 1–10; “>10 (Relative abundance)” indicates a relative abundance > 10. “≤1 (Sequence number)” indicates a sequence number ≤ 1; “1–100 (Sequence number)” indicates a sequence number 1–100; “100–1000 (Sequence number)” indicates a sequence number 100–1000; “1000–2000 (Sequence number)” indicates a sequence number 1000–2000; “2000–3000 (Sequence number)” indicates a sequence number 2000–3000; “70,000–80,000 (Sequence number)” indicates a sequence number 70,000–80,000; “90,000–100,000 (Sequence number)” indicates a sequence number 90,000–100,000.

## Data Availability

The datasets used and/or analyzed during the current study are available from the corresponding author on reasonable request.

## Abbreviations

*Up*	*Ureaplasma parvum*
NGS	next-generation sequencing
mNGS	metagenomic next-generation sequencing
IRB	Institutional Review Board
BALF	bronchoalveolar lavage fluid
*CMV*	*Cytomegalovirus*
NE	neutrophils
WBC	white blood cells
*PA*	*Pseudomonas aeruginosa*
